# Efficacy and safety of *Tripterygium wilfordii* hook F for chronic urticaria: a systematic review and meta-analysis

**DOI:** 10.1186/s12906-018-2305-7

**Published:** 2018-08-31

**Authors:** Liu Liu, Huaibo Zhao, Xiaoying Sun, Qi Zheng, Ying Luo, Yi Ru, Ying Zhang, Xi Chen, Bo Zhu, Chengqian Yin, Bin Li, Xin Li

**Affiliations:** 1 Department of Dermatology, Yueyang Hospital of Integrated Traditional Chinese and Western Medicine, Shanghai University of Traditional Chinese Medicine, Shanghai, 200437 China; 2 Institute of Dermatology, Shanghai Academy of Traditional Chinese Medicine, Shanghai, 201203 China; 3 Department of Dermatology, Shaanxi Hospital of Traditional Chinese Medicine, Xi’an, 710003 China; 4 Department of Pharmacology & Experimental Therapeutics, Boston University School of Medicine, Boston, MA 02118 USA

**Keywords:** Chronic urticaria, *Tripterygium wilfordii* hook F, *Tripterygium* agents, Systematic review, Meta-analysis

## Abstract

**Background:**

The first-line agents comprising antihistamines for chronic urticaria, are not completely satisfactory. *Tripterygium wilfordii* Hook F (TwHF), a Chinese herb, has been developed into several *Tripterygium* agents and have definite effects on autoimmune and inflammatory diseases. In chronic urticaria, however, their values of practical application remain unclear. The aim of this study was to investigate the efficacy and safety of TwHF in patients with chronic urticaria.

**Methods:**

Several databases were systematically searched including PubMed, Embase, Cochrane Central Register of Controlled Trials, China Network Knowledge Infrastructure, Chinese Scientific Journals Database, Wan Fang Database, and Chinese Biomedicine. Randomized controlled trials comparing antihistamines with TwHF or *Tripterygium* agents in combination with antihistamines were included. Revman5.3 was utilized to calculate risk ratios (RR) with 95% confidence intervals (CI). This study was registered with PROSPERO, number CRD42018091595.

**Results:**

Twenty-one trials with 2565 participants were included in this analysis. Meta-analysis showed that, when antihistamines were combined with TwHF and *Tripterygium* agents, the curative effect in cases of chronic urticaria was superior to that of antihistamines alone (RR: 1.40; 95% CI: 1.33–1.46). The incidence rates of gastrointestinal disorder (RR: 2.91; 95% CI: 1.70–4.99) and menstrual disorder (RR: 6.00; 95% CI: 1.79–20.13) in drug combination groups were higher than those in controls, while other adverse events were similar between the two groups. After treatment, Dermatology Life Quality Index (RR: 1.23; 95% CI: 1.09–1.40), quality of sleep (RR: 1.50; 95% CI: 1.07–2.12), and daily activity (RR: 1.49; 95% CI: 1.25–1.78) were all improved. Furthermore, drug combination groups demonstrated less relapse (RR: 0.34; 95% CI: 0.25–0.45).

**Conclusions:**

TwHF and *Tripterygium* agents, in combination with antihistamines, appear to be more effective than antihistamines alone. Nevertheless, adverse events cannot be ignored. Large sample, multi-center, high-quality clinical studies are needed to verify the exact effects and safety of TwHF and *Tripterygium* agents in treatment of chronic urticaria.

**Electronic supplementary material:**

The online version of this article (10.1186/s12906-018-2305-7) contains supplementary material, which is available to authorized users.

## Background

Urticaria is a recurrent dermatosis, characterized by spontaneous wheals, angioedema, or both. Generally, chronic urticaria is defined as occurrence of urticaria for longer than 6 weeks [1]. It affects 0.5–1% of individuals and reduces the quality of life significantly [2]. Histamine release, driven by mast cells, is regarded as the primary feature in chronic urticaria, resulting in the presence of wheals and flare [3]. Thus, treatment with antihistamines plays a crucial role in chronic urticaria. First-line agents in the routine management of chronic urticaria comprise second-generation antihistamines, such as mizolastine, levocetirizine, and desloratadine [1]. However, high recurrence rate and drug resistance are an ongoing matter of concern. Thus, better therapy is required.

*Tripterygium wilfordii* Hook F (TwHF) is a vital Chinese herbs belonging to the Celastraceae family. In China, four species of the genus *Tripterygium* are prevalent, including *Tripterygium wilfordii* Hook F, *Tripterygium hypoglaucum* (levl.) Hutch (THH), *Tripterygium regelii* Sprague and Takeda and *Tripterygium forrestii* Loes. Molecular analyses have indicated *Tripterygium wilfordii* and *Tripterygium hypoglaucum* are not distinct, while *Tripterygium regelii* is considered as a separate species [4]. Their effects include anti-inflammation, antianaphylaxis, and immunosuppression. In 2007, triptolide and celastrol, the main components of TwHF, along with artemisinin, capsaicin, and curcumin, were listed as the most promising natural traditional medicines [5]. Over the past few decades, several kinds of *Tripterygium* agents extracted from the root bark of this herb, such as Glucosidorum Tripterygll Totorum tablets (GTT), tripterygium glycosides tablets (TG), and *Tripterygium hypoglaucum* (levl.) Hutch tablets (THH), have been developed and used for treating autoimmune and inflammatory diseases in China; these diseases include rheumatoid arthritis [6], diabetic nephropathy [7], purpura nephritis [8], and urticaria [9, 10]. Currently, increasing evidence has reported that TwHF combined with antihistamine for chronic urticaria sufferers is satisfactory. Nevertheless, the curative effect and safety of these agents in urticaria is not clear. It is essential to evaluate the trials in this subject in order to effectively conduct the practice of medicine. Hence, we undertook this systematic review of randomized controlled trials (RCTs) to examine the effectiveness of TwHF and *Tripterygium* agents, in combination with antihistamines, in chronic urticaria.

## Methods

The review protocol was registered in the PROSPERO database before the start of the review process (CRD42018091595). This study was performed according to the Cochrane Handbook for Systematic Reviews of Interventions [11] and is presented in accordance with the Preferred Reporting Items for Systematic Reviews and Meta-analyses (PRISMA) guidelines (Additional file 1: Table S1) [12].

### Search trials

We searched databases from their inception dates through February 25, 2018, in order to determine the efficacy and safety of TwHF in chronic urticaria. Included databases were as follows: PubMed, Embase, Cochrane Central Register of Controlled Trials (CENTRAL), China Network Knowledge Infrastructure (CNKI), China Science and Technology Journal Database (VIP), Wan Fang Database, and Chinese Biomedicine (CBM). We combined keywords from MeSH headings with self-generated key words to identify studies, using unrestricted language. An additional search was performed on relevant websites, including Clinical Trials (http://www.clinicaltrials.gov) and the Chinese Clinical Trial Registry (http://www.chictr.org.cn/index.aspx), to identify similar studies.

### Inclusion criteria

RCTs were included if they met the following inclusion criteria: (1) RCTs with explicit diagnostic standards or meeting the criteria for chronic urticaria; (2) RCTs, regardless of race and gender, comprising patients ranging from 12 to 73 years of age; (3) RCTs comparing either TwHF or *Tripterygium* agents with antihistamines.

### Outcomes

Primary outcomes included symptom scores, such as the Level Four Score method (LFS), Urticaria Activity Score (UAS), and Symptom Score Reduce Index (SSRI). Adverse events (ADE) were also included. Secondary outcome measurements included Dermatology Life Quality Index (DLQI) and Recurrence Rate (RER).

### Extraction criteria

Two reviewers (Y.R, Y.Z) independently determined the following information in each study: lead author, publication year, sample size (treatment group, control group), typical course of disease, interventions (treatment group, control group), outcome criteria, and adverse reactions. In case of differing opinions, consensus was reached after a discussion. We excluded urticaria patients with specific etiologies, such as hereditary angioedema. Control groups using any kinds of *Tripterygium* agents were excluded. If the literature did not provide control group at any point in the analysis, the study was excluded. Additionally, interventions with unclear dosing regimens and partially recorded adverse events were excluded. We used the most recent article in cases of republication.

### Risk-of-Bias assessments

To analyze included RCTs, two reviewers (Q.Z, Y.L) assessed the Risk-of-Bias independently, using The Cochrane Risk of Bias Tool [11]. Each term was divided into three grades—low risk, high risk, and unclear risk—based on the following criteria: (1) Random sequence generation; (2) Allocation concealment; (3) Blinding of participants and personnel; (4) Blinding of outcome assessment; (5) Incomplete outcome data; (6) Selective reporting; (7) Other bias. Disagreements were discussed between the two reviewers, and if were unresolved, a third reviewer (X.L) was added to the discussion until a consensus was reached.

### Statistical analysis

Revman5.3 software, provided by the Cochrane Collaboration, was utilized to assess dichotomous data, using risk ratio (RR), and continuous data, using mean difference (MD) and standard mean difference (SMD), with 95% confidence intervals. Heterogeneity was tested using I^2^ statistics. If there was homogeneity (*P* > 0.1, I^2^ < 50%) in the results, we used a fixed effects model. Otherwise, we used a random effects model. Subgroups analyses were performed to avoid heterogeneity. A funnel plot was used to analyze bias.

## Results

### Included studies and the characteristics

**Fig. 1:**
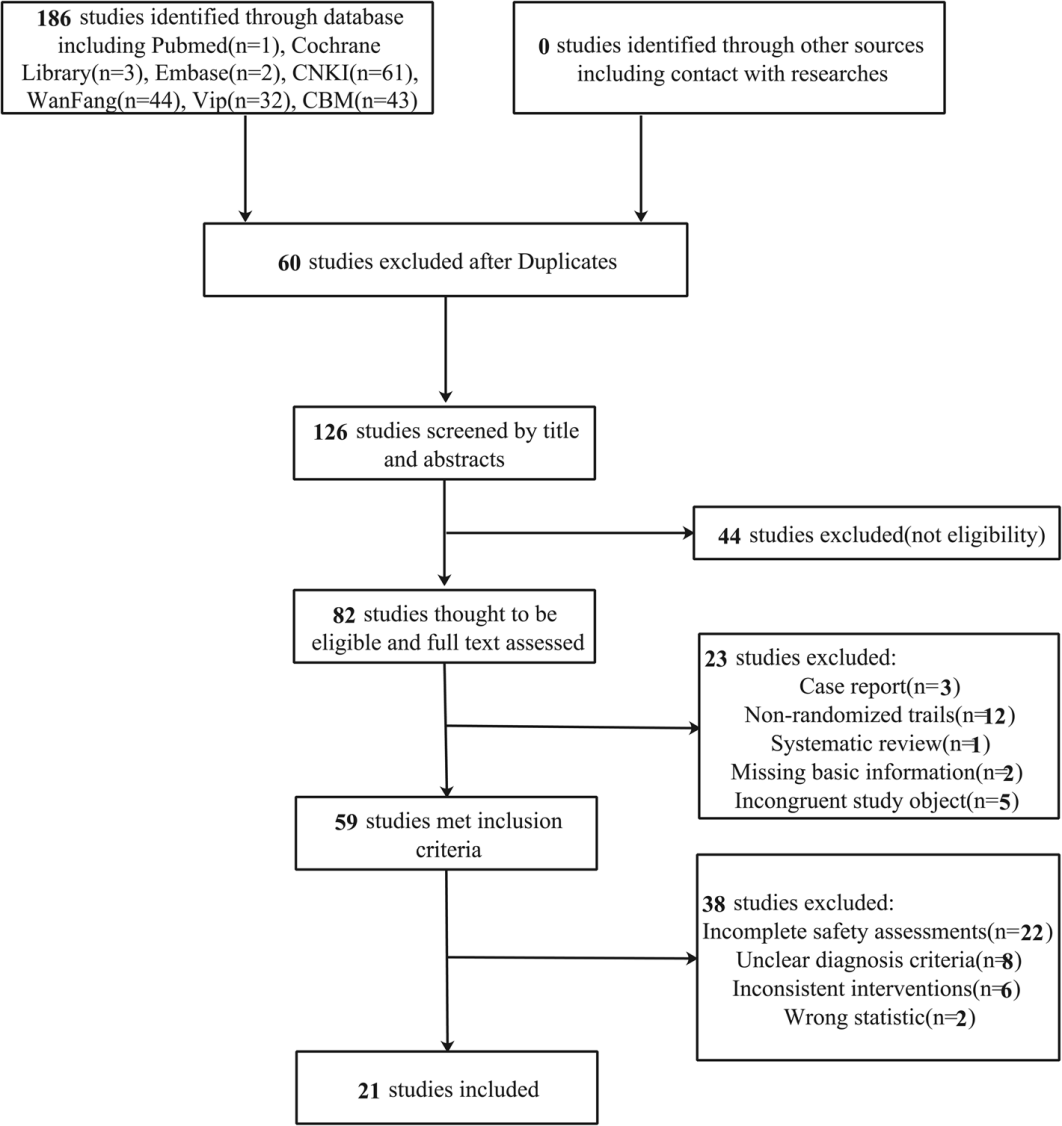
Literature search and study selection flowchart

After initial retrieval from six databases, 186 citations were identified. Sixty duplicate articles were excluded; an additional 126 articles were excluded after reading titles and abstracts. After the full-text reading of the resulting 82 articles, 21 studies [9, 13–32] met our inclusion criteria (Fig. 1).

Altogether, 2565 participants were included in the 21 studies of this analysis, ranging from 12 to 73 years of age. RCTs were published in English and Chinese; all originated from China. All trials utilized diagnostic criteria. Twelve trials [14, 16, 19–22, 25, 27, 29–32] were in accordance with dermatology monographs in China. One trial [23] applied guidance for the diagnosis and treatment of urticaria (2007). Four trials [9, 13, 17, 24] mentioned diagnostic criteria, while the remaining four trials [15, 18, 26, 28] met diagnostic criteria for urticaria.

The included trials were neither multicentered nor reported syndrome differentiation as illustrated in traditional Chinese Medicine. The basic characteristics of involved studies are presented in Table 1. In treatment groups, 11 trials [15, 17, 19, 23, 25–28, 30–32] used GTT, six trials [9, 13, 16, 21, 22, 29] used TG, three trials [18, 20, 22] used THH, and one trial [14] used TwHF. The control groups used different types of second-generation antihistamines. Three of these trials [9, 13, 22] used a combination with mizolastine, five trials [9, 14, 18, 19, 24] with cetirizine, nine trials [15–17, 25, 27, 28, 30–32] used desloratadine, while one trial [9] utilized loratadine. Two trials [23, 29] used ebastine, one trial [20] used terfenadine, and two trials [21, 26] used fexofenadine. Duration of treatment ranged from 4 to 12 weeks, and the courses of 19 trials [9, 14–21, 23–32] were 28 d.

**Table 1:** Characteristics of included trials

Study (author/year)	Sample size (I/C)	Average courses ($\overline{\mathrm{X}}$[SD])		Interventions		Course of treatment	Used instruments in the study
		T	C	T	C		
Pi 2006 [13]	21/22	1.30 [2.03] y		TG + Mizolastine	Mizolastine	≤12 w	LFS, SSRI, ADE
Zhang 2007 [14]	86/82	0.85 y		TwHF + Levocetirizine	Levocetirizine	4 w	LFS, SSRI, ADE
Bao 2008 [15]	87/80	1.60 y		GTT + Desloratadine	Desloratadine	4 w	LFS, SSRI, ADE
Xu2009 [16]	54/54	1.08 y	1.17 y	TG + Desloratadine	Desloratadine	4 w	LFS, SSRI, ADE, RER
Wei 2010 [17]	90/90	6.25 y	6.20 y	GTT + Desloratadine	Desloratadine	4 w	LFS, SSRI, ADE, DLQI
Zhong 2011 [18]	40/38	16.75 y	16.25 y	THH + Cetirizine Hydrochloride	Placebo + Cetirizine Hydrochloride	4 w	LFS, TER, ADE
Liu 2011 [19]	98/90	16.20 [0.96] y		GTT + Levocetirizine Hydrochloride	Levocetirizine Hydrochloride	4 w	LFS, SSRI, ADE
Qian 2011 [9]	180/180	6.72 [3.47] y	6.51 [3.76] y	TG + Loratadine; TG + Mizolastine; TG + Cetirizine	Loratadine; Mizolastine; Cetirizine	4 w	LFS, UAS,S SRI, ADE, DLQI, RER
Yu 2011 [20]	48/47	1.7 y		THH + Terfenadine	Terfenadine	4 w	SA, ADE
Lu 2012 [21]	40/40	3.20 [1.60] y	3.20 [1.20] y	TG + Fexofenadine hydrochloride	Fexofenadine hydrochloride	4 w	LFS, SSRI, ADE
Zhou 2012 [22]	60/60	3.97 [2.97] y	3.45 [2.72] y	THH + Mizolastine + Ketotifen^a^	Mizolastine + Ketotifen^a^	8 w	UAS, SSRI, ADE
Fan 2013 [23]	43/41	2.70 y	2.77 y	GTT + Ebastine	Ebastine	4 w	LFS, SSRI
Liu 2013 [24]	89/90	≥0.13 y		TG + Cetirizine Hydrochloride	Cetirizine Hydrochloride	4 w	LFS, VAS, SSRI, ADE, RER
Zheng 2013 [25]	37/37	≥0.13 y		GTT + Desloratadine	Desloratadine	4 w	LFS ,SSRI, ADE,DLQI, RER
Chen 2014 [26]	45/46	3.70 [1.30] y	3.90 [1.10] y	GTT + Fexofenadine	Fexofenadine	4 w	UAS, VAS, LFS, ADE
Wang 2014 [27]	32/32	≥0.13 y		GTT + Desloratadine	Desloratadine	4 w	SA, ADE
Zhou 2014 [28]	60/60	≥0.13 y		GTT + Desloratadine	Desloratadine	4 w	LFS, SSRI, ADE, RER
Li 2016 [29]	36/34	≥0.13 y		TG + Ebastine	Ebastine	4 w	SA, ADE
Liu 2016 [30]	60/60	0.53 [0.27] y	0.58 [0.33] y	GTT + Desloratadine	Desloratadine	4 w	LFS, SSRI, ADE, DLQI
Chen 2017 [31]	45/45	0.56 [0.32] y	0.53 [0.27] y	GTT + Desloratadine	Desloratadine	4 w	SA, ADE
Tao 2017 [32]	48/49	2.46 [0.52] y	2.53 [0.63] y	GTT + Desloratadine	Desloratadine	4 w	LFS, SSRI, ADE, DLQI, RER

^a^Administered ketotifen for 4 weeks

*TwHF Tripterygium wilfordii* Hook F, *GTT* Glucosidorum Tripterygll Totorum, *TG* Tripterygium Glycosides, *THH Tripterygium hypoglaucum* (levl.) Hutch, *LFS* Four-level Score Method, *UAS* Urticaria Activity Score, *SSRI* Symptom Score Reduce lndex, *ADE* Adverse Events, *DLQI* Dermatology Life Quality Index, *RER* Recurrence Rate, *TER* Total Effective Rate, *VAS* Visual Analogue Scales, *SA* Symptom Assessment

Regarding outcomes, thirteen studies [13–17, 19, 21, 23–25, 28, 30, 32] used LFS and SSRI to describe findings, while two trials [9, 22] used UAS and SSRI. One trial [18] measured LFS and Total Efficacy Rate (TER) and another [26] only evaluated UAS. The results of four trials [20, 27, 29, 31] were described by symptom assessments.

### Risk of bias

**Fig. 2:**
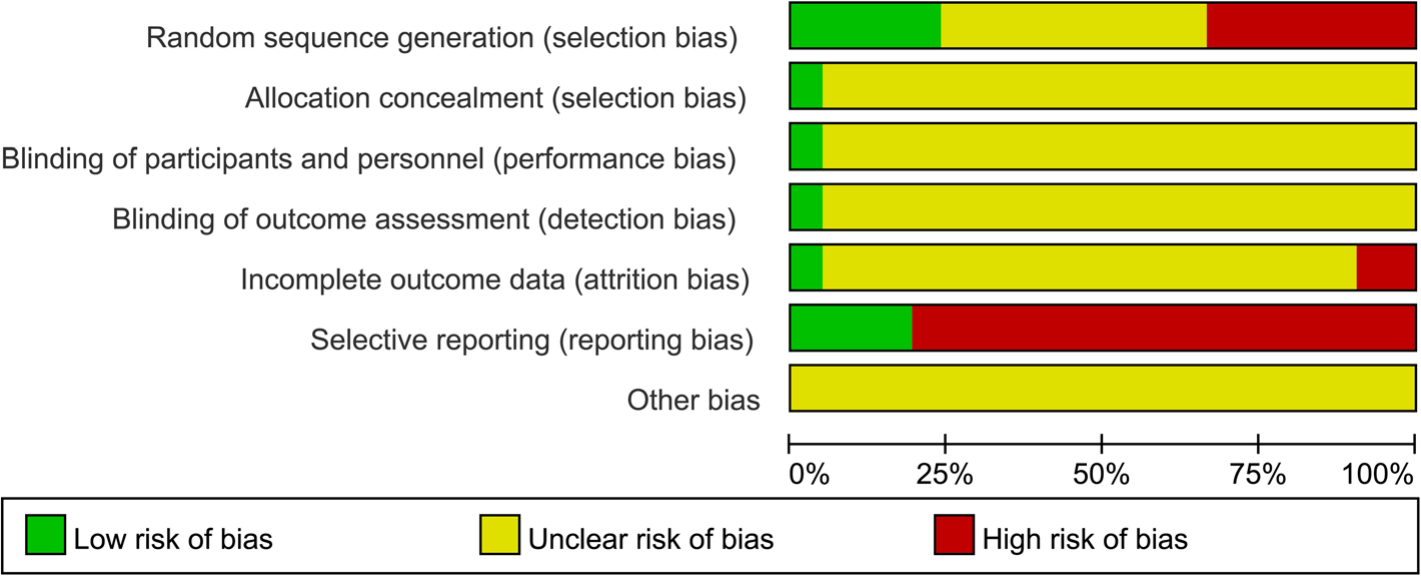
Risk of bias graph

**Fig. 3:**
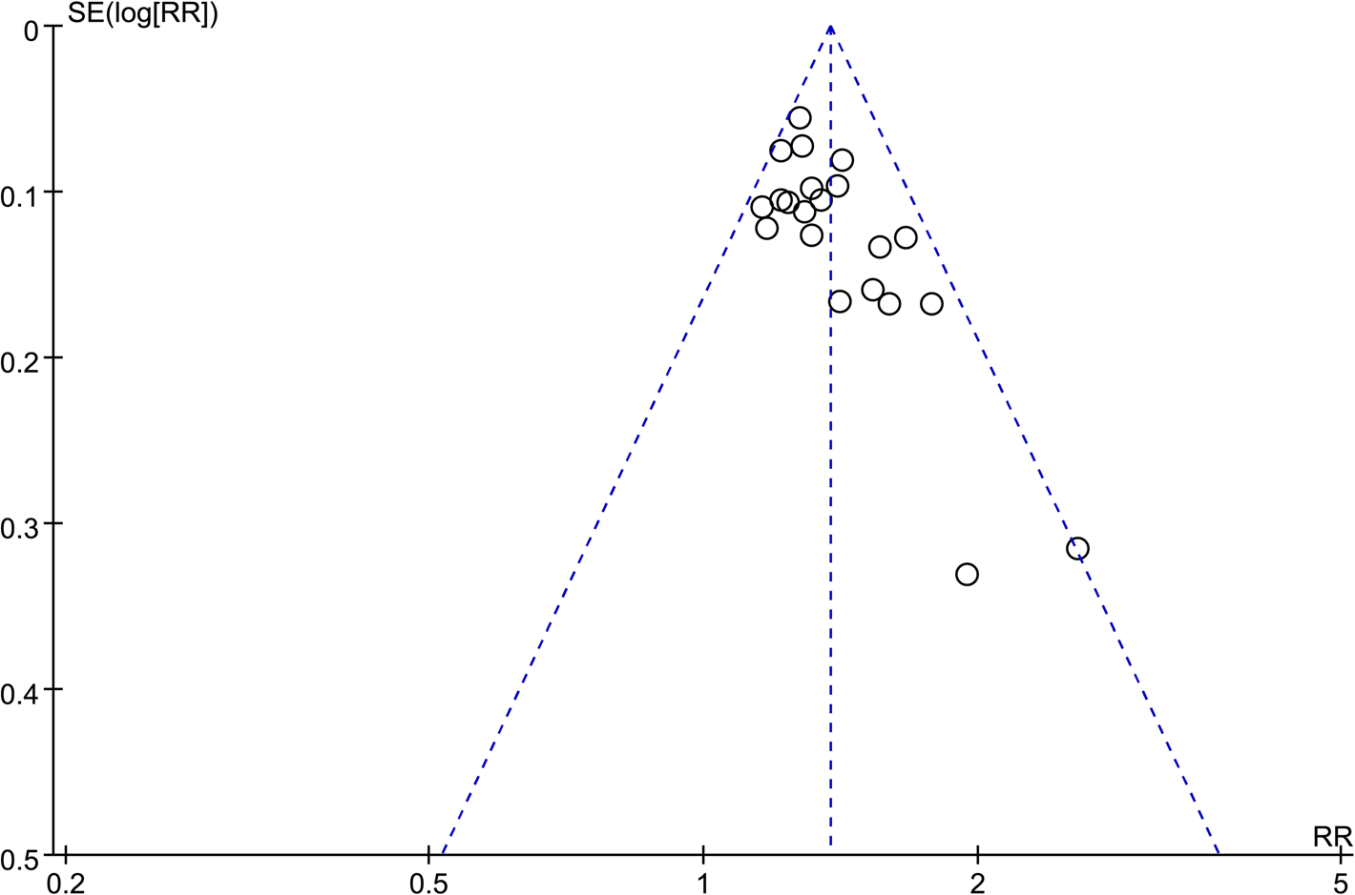
Funnel plot graph

Ten of the included trials [16–19, 22, 24–26, 28, 32] reported random sequence generation, five of which [16, 17, 19, 22, 24] were based on treatment order and the remaining five [18, 25, 26, 28, 32] were based on tables of random numbers. Only one article [18] mentioned allocation concealment, blinding method, whereas the others did not. Patients in three trials [13, 17, 18] withdrew from the studies and one trial [18] reported intentional (ITT) analysis, while the remaining did not. Three trials [9, 25, 32] reported complete outcomes. None of the trials reported other bias (Fig. 2). Asymmetric distribution of the trials is presented in a funnel plot (Fig. 3), which implies low-quality methodology and suggests that a publication bias may exist. The small sample size may be a major reason for this possible bias.

### Primary outcomes

#### Efficacy evaluation

**Fig. 4:**
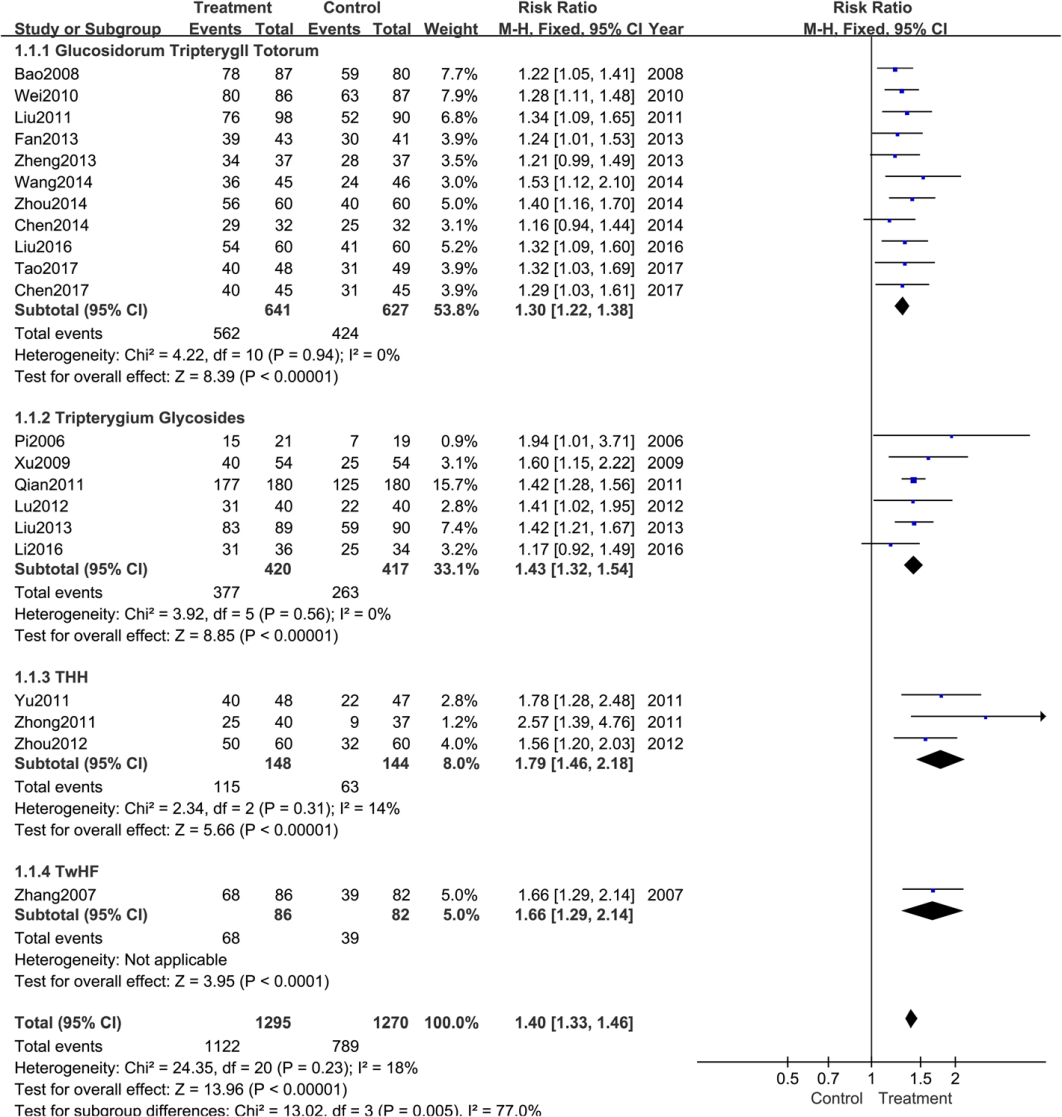
Meta-analysis of the effectiveness rate of *Tripterygium* preparations combined with antihistamines versus antihistamines. CI indicates confidence interval

Effectiveness rate was all evaluated in interventions and controls in 21 trials. According to the variety of *Tripterygium* agents, all trails were divided into four groups to perform subgroup analyses (Fig. 4). The pooled results indicated that antihistamines combined with GTT (RR: 1.30; 95% CI: 1.22–1.38; *P* < 0.001; fixed model; I^2^ = 0%; eleven trials), TG (RR: 1.43; 95% CI: 1.32–1.54; P < 0.001; fixed model; I^2^ = 0%; six trials), THH (RR: 1.79; 95% CI: 1.46–2.18; *P* < 0.001; fixed model; I^2^ = 14%; three trials), and TwHF (RR: 1.66; 95% CI: 1.29–2.14; *P* < 0.001; fixed model; one trial) were superior to antihistamines alone.

#### Adverse events

**Fig. 5:**
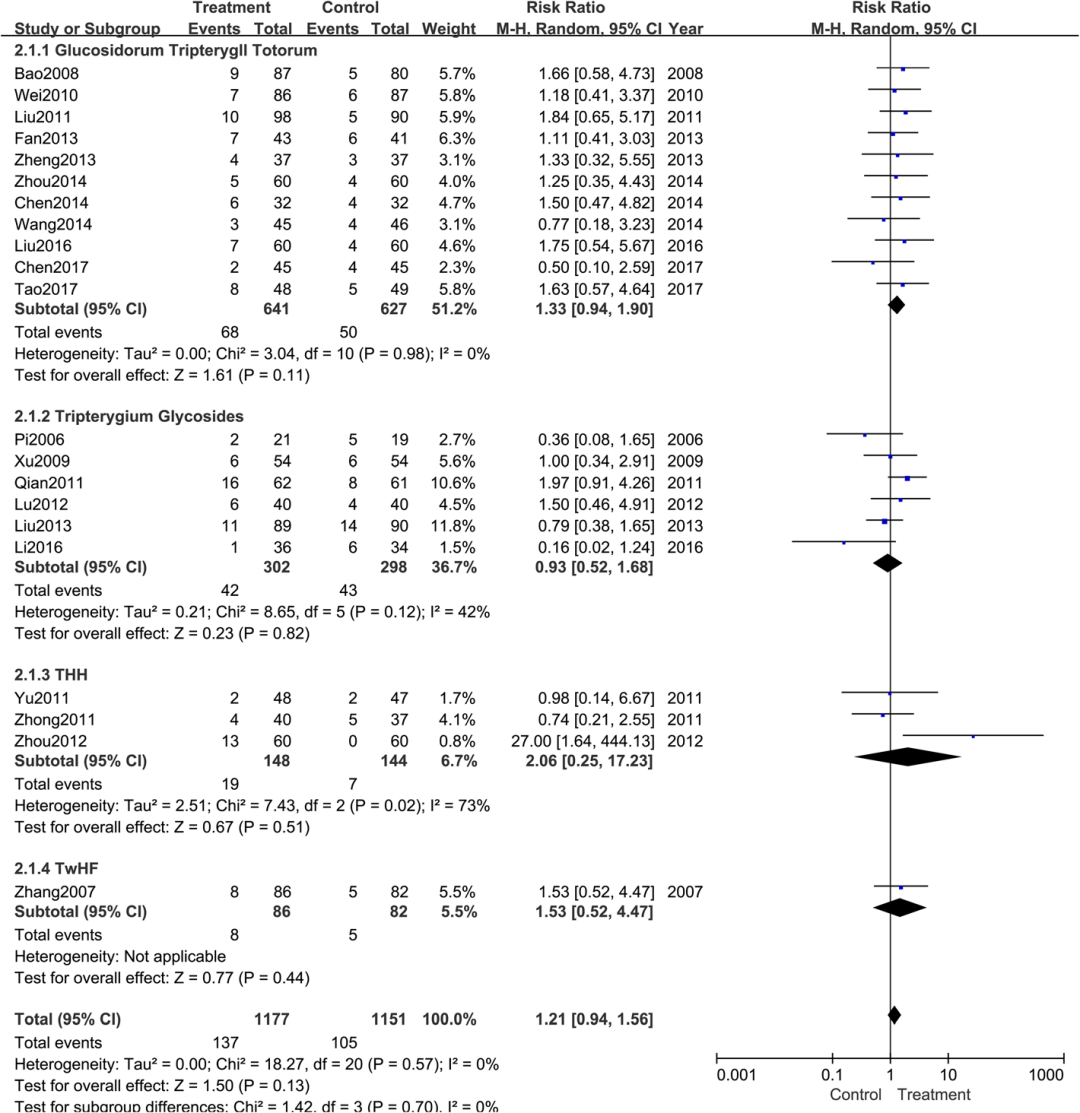
Meta-analysis of adverse events of *Tripterygium* preparations combined with antihistamines versus antihistamine. CI indicates confidence interval

Each trial reported adverse events (Table 2). Subgroup analysis was performed according to different diversities of *Tripterygium* agents and different symptoms. As presented in Fig. 5, interventions with GTT (RR: 1.33; 95% CI: 0.94–1.89; *P* = 0.11; random model; I^2^ = 0%; eleven trials), TG (RR: 0.93; 95% CI: 0.52–1.68; *p* = 0.82; random model; I^2^ = 42%; six trials), THH (RR: 2.06; 95% CI: 0.25–17.23; *P* = 0.51; random model; I^2^ = 73%; three trials), and TwHF (RR: 1.53; 95% CI: 0.52–4.47; *P* < 0.001; random model; one trial) were similar to control groups. All the trails reported adverse events including drowsiness, gastrointestinal disorder, menstrual disorders, abnormal liver function, etc. Details are shown in Table 2.

**Table 2:** Adverse events of included trials

Study	Adverse events	
(author/year)	Intervention	Control
Pi 2006 [13]	Two menstrual disorders	Five drowsiness
Zhang 2007 [14]	Eight drowsiness, sleepiness and fatigue, gastrointestinal disorder, menstrual disorders	Five drowsiness, dry mouth, dizzy
Bao 2008 [15]	Seven drowsiness, sleepiness and fatigue, dry mouth; two gastrointestinal disorder, menstrual disorders	Five drowsiness, sleepiness and fatigue, dry mouth
Xu 2009 [16]	Five drowsiness, sleepiness and fatigue, dry mouth; one irregular menses, abnormal liver function	Four drowsiness, dizzy; two gastrointestinal disorder
Wei 2010 [17]	Three drowsiness, sleepiness and fatigue, dry mouth; two gastrointestinal disorder; one headache, dizzy; one menstrual disorders, abnormal liver function	Three drowsiness, sleepiness and fatigue, dry mouth; two gastrointestinal disorder; two headaches, dizzy;
Zhong 2011 [18]	Four cases including drowsiness, sleepiness and fatigue, gastrointestinal disorder	Five cases including drowsiness, sleepiness and fatigue, gastrointestinal disorder
Liu 2011 [19]	Two drowsiness; one sleepiness and fatigue; three gastrointestinal disorder; one bloating; two menstrual disorders	Two drowsiness; two dry mouth; one dizzy;
Qian 2011 [9]	Four gastrointestinal disorder; three dizzy; three insomnias; three palpitation; three alopecia	Seven drowsiness; one dizzy; one general malaise
Yu 2011 [20]	One gastrointestinal disorder; one dizzy	Two headaches, gastrointestinal disorder
Lu 2012 [21]	Two drowsiness; one sleepiness and fatigue; two gastrointestinal disorder; one dry mouth	One drowsiness; one sleepiness and fatigue; two dry mouth
Zhou 2012 [22]	10 gastrointestinal disorder; three abnormal liver functions	No significant side effects.
Fan 2013 [23]	Two drowsiness; three sleepiness and fatigue; two dizzy	Two drowsiness; two sleepiness and fatigue; two dizzy
Liu 2013 [24]	Nine drowsiness; two dry mouth	11 drowsiness; three dry mouth
Zheng 2013 [25]	One drowsiness; one sleepiness and fatigue; one gastrointestinal disorder; one headache	One dizzy; one gastrointestinal disorder; one dry mouth
Chen 2014 [26]	Two drowsiness, sleepiness and fatigue; four gastrointestinal disorder;	Four drowsiness, sleepiness and fatigue;
Wang 2014 [27]	One drowsiness; one sleepiness and fatigue; one gastrointestinal disorder	One drowsiness; one sleepiness and fatigue; two gastrointestinal disorder
Zhou 2014 [28]	Two drowsiness, sleepiness and fatigue, dry mouth; one gastrointestinal disorder; one headache, dizzy; one abnormal liver function	Two drowsiness, sleepiness and fatigue, dry mouth; one gastrointestinal disorder; one headache, dizzy;
Li 2016 [29]	One dizzy	Three drowsiness; one sleepiness and fatigue; one dizzy
Liu 2016 [30]	Three drowsiness; two gastrointestinal disorder; two dizzy	One drowsiness; two gastrointestinal disorder; one dizzy
Chen 2017 [31]	One headache, dizzy; one gastrointestinal disorder	One drowsiness; two headaches, dizzy; one gastrointestinal disorder
Tao 2017 [32]	Three drowsiness, sleepiness and fatigue; five gastrointestinal disorder;	Five drowsiness, sleepiness and fatigue;

The different symptoms of adverse events are shown in Fig. 6. Zhong et al. [18] reported nine adverse events in two groups; the most common was gastrointestinal disorder. Subgroup analysis revealed that gastrointestinal disorder [9, 14–17, 19–21, 25–28, 30–32] occurred in 47 participants. The pooled results showed that a higher number of adverse events occurred in drug combination groups than in control groups (RR: 2.91; 95% CI: 1.70–4.99; *P* < 0.001; fixed model; I^2^ = 13%; sixteen trials). Six trials [13–17, 19] had 16 patients with irregular menses. Subgroup analyses showed that there was a significant difference between the two groups (*p* = 0.004). After treatment with TwHF or any other *Tripterygium* agents plus antihistamines, the risk of irregular menses was improved for women, whereas control groups did not exhibit this adverse event (RR: 6.00; 95% CI: 1.79–20.13; *P* = 0.004; fixed model; I^2^ = 0%).

**Fig. 6:**
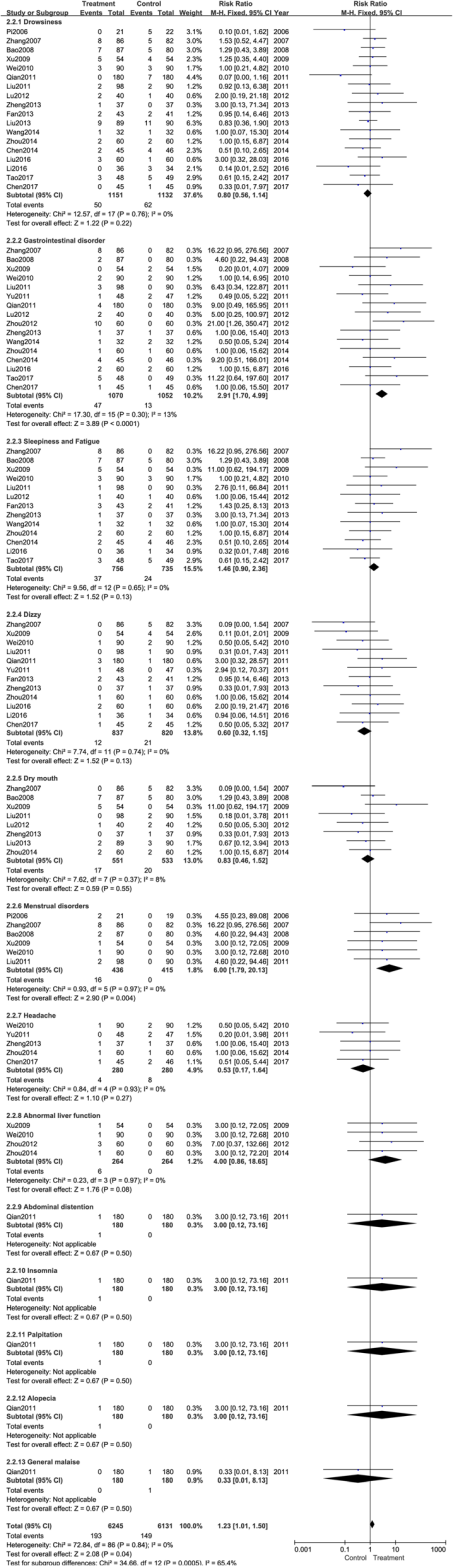
Meta-analysis of different symptoms of adverse events of *Tripterygium* preparations combined with antihistamines versus antihistamine. CI indicates confidence interval

In addition to the above two types of adverse events, the most common adverse event was drowsiness, observed in 18 trials [9, 13–17, 19, 21, 23–32] with 50 patients (RR: 0.80; 95% CI: 0.56–1.14; *P* = 0.22; fixed model; I^2^ = 0%; eighteen trials). Thirty-seven patients experienced sleepiness and fatigue [14–17, 19, 21, 23, 25–29, 32] (RR: 1.46; 95% CI: 0.90–2.36; *P* = 0.13; fixed model; I^2^ = 0%; thirteen trials). Moreover, 12 participants in 12 trials [9, 14, 16, 17, 19, 20, 25–32] (RR: 0.60; 95% CI: 0.32–1.15; *P* = 0.13; fixed model; I^2^ = 0%) experienced dizziness. Eight trials [14–17, 19, 21, 22, 25, 28] (RR: 0.83; 95% CI: 0.46–1.52; *P* = 0.55; fixed model; I^2^ = 8%) reported dry mouth. Five trials [17, 20, 25, 28, 31] (RR: 0.53; 95% CI: 0.17–1.64; *P* = 0.27; fixed model; I^2^ = 0%) reported headache. Four trials [16, 17, 22, 28] reported abnormal liver function (RR: 4.00; 95% CI: 0.86–18.65; *P* = 0.08; fixed model; I^2^ = 0%). Qian et al. [9] reported abdominal distention (RR: 3.00; 95% CI: 0.12–73.16; *P* = 0.50; fixed model), palpitation (RR: 3.00; 95% CI: 0.12–73.16; *P* = 0.50; fixed model), alopecia (RR: 3.00; 95% CI: 0.12–73.16; *P* = 0.50; fixed model), and insomnia (RR: 3.00; 95% CI: 0.12–73.16; *P* = 0.50; fixed model) when treated with *Tripterygium* agents.

### Secondary outcomes

#### Dermatology life quality index (DLQI)

**Fig. 7:**
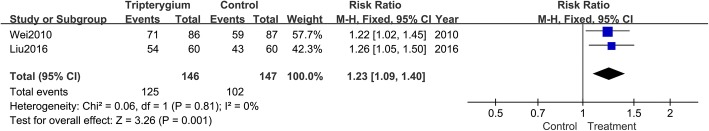
Meta-analysis of Dermatology Life Quality Index. CI indicates confidence interval

DLQI is an important content in urticaria evaluation; five trials [9, 17, 25, 30, 32] have described it. One study [25] measured quality of sleep and daily activity in the place of DLQI. Two studies [9, 32] reported scores, while the remaining [17, 30] reported affected cases. As shown in Fig. 7, DLQI (RR: 1.23; 95% CI: 1.09–1.40; *P* = 0.001; fixed model; I^2^ = 0%; two trials) was obviously improved in the intervention groups.

#### Quality of sleep

**Fig. 8:**
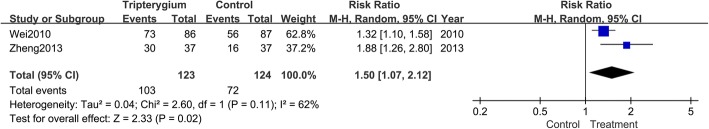
Meta-analysis of Quality of sleep. CI indicates confidence interval

Three trials [17, 25, 32] measured quality of sleep as an outcome. One study [32] showed that the scores in the drug combination group were lower than in controls, which indicates better sleep intervention. Meta-analysis showed that in the other two studies, *Tripterygium* agents combined with antihistamines were significantly better than antihistamines alone (RR: 1.50; 95% CI: 1.07–2.12; *P* = 0.02; random model; I^2^ = 62%; two trials) (Fig. 8).

#### Quality of daily activity

**Fig. 9:**
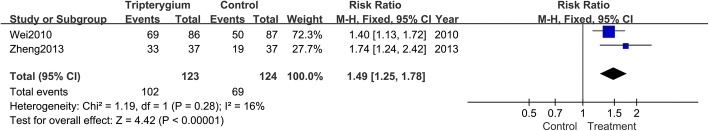
Meta-analysis of Quality of daily activity. CI indicates confidence interval

Three trials [17, 25, 32] used quality of daily activity as an evaluation method. One study [32] showed that compared with antihistamine alone, daily activity in the drug combination group was of a higher quality. Results of the remaining trials showed obvious improvements in the treatment groups, when compared with control groups (Fig. 9) (RR: 1.49; 95% CI: 1.25–1.78; *P* < 0.001; fixed model; I^2^ = 16%; two trials).

#### Recurrence rate

**Fig. 10:**
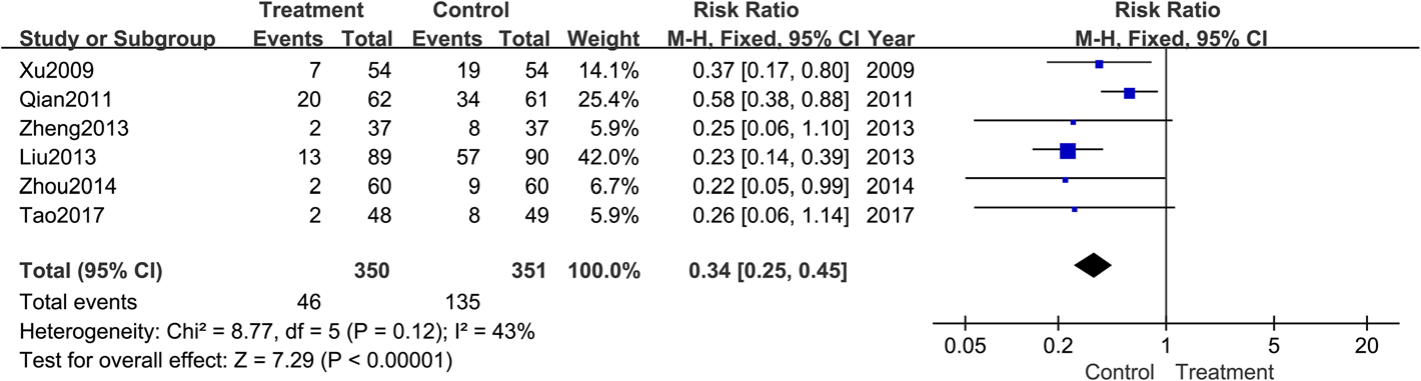
Meta-analysis of recurrence rates during follow-up. CI indicates confidence interval

Six trials [9, 16, 24, 25, 28, 32] utilized recurrence rate as a secondary outcome. Subgroup analysis showed that patients treated with *Tripterygium* agents had lower recurrence rates, that did patients without the treatment (RR: 0.34; 95% CI: 0.25–0.45; *P* < 0.001; fixed model; I^2^ = 43%; six trials) (Fig. 10).

## Discussion

This systematic review, including 21 trials, evaluated the efficacy and safety of *Tripterygium* in the treatment of chronic urticaria. Although the quality of these studies were not highly satisfactory, the results showed that, compared to treatment with antihistamines alone, the combination with TwHF or *Tripterygium* agents improved both, the symptoms and quality of life; even the recurrence rates were lower. However, adverse events could not be ignored. There was marked difference between the two groups in terms of gastrointestinal reactions and menstrual disorders. A total of 47 participants in the drug combination groups experienced gastrointestinal discomfort. Sixteen female patients in these groups experienced menstrual disorders, while those in control groups did not. For men who had not procreated, one trial [22] evaluated sperm motility in the intervention group and found an influence on sperm motility, which suggests that there may be a certain risk to the reproductive system.

In China, Wen et al. [33] performed a systematic evaluation in 2015 of GTT combined with antihistamines for chronic idiopathic urticaria, including 21 trials. Results of their systematic review were consistent with our findings that combined GTT with antihistamines had an obvious effect. However, as increasing evidence emerges every year, systematic reviews should be updated in a timely fashion. In this review, we added literature published in the most recent two years and formulated a set of rigorous inclusion and exclusion criteria. Furthermore, we have included all types of *Tripterygium* preparations.

Urticaria is known as a mast cell-driven disease, the key aspect of which is degranulation of mast cells with release of histamines and synthesis of inflammatory cytokines, causing an increase in capillary permeability and leading to edema of the dermis. Although antihistamines are effective, there are reports of drug resistance, high recurrence rates, and several side effects in RCTs. TwHF, as a significant traditional Chinese herbal medicine, has effects on numerous symptoms, according to basic theory of Chinese medicine. Pharmacological studies demonstrated that TwHF plays a significant role in antitumor, anti-inflammatory, and immune suppression mechanisms [34]. Chronic urticaria is classically thought to have a basis in autoimmunity and mast cells are vital to it. It has been reported by Liu et al. [35] and Yao et al. [36] that tripterine, a primary component in TwHF, has the ability to inhibit the degranulation of mast cells and histamine release. Although the role of TwHF in chronic urticaria remains unclear, multiple studies have reported stronger effects when treated with TwHF and its agents.

Nevertheless, adverse events are always a focus of concern. It is generally known that the active ingredients in TwHF are also toxic components that may be harmful to the liver, kidneys, reproductive tissues, and immune tissues. Although *Tripterygium* agents are developed with attenuation measures, toxic effects are inevitable. In this meta-analysis, we found that patients in drug combination groups were more likely to experience gastrointestinal disorders and altered menstruation. Gastrointestinal disorder is a serious adverse event in trials involving TwHF and *Tripterygium* agents, the incidence rate of which is 25.2% [37]. Even within the normal dose range, the main manifestations are nausea, vomiting, and bloating. Yang et al. [38] treated NIH mice and Sprague-Dawley rats with different doses and found that they all exhibited pathological changes in the gastrointestinal tract in a dose-dependent manner. Over time, gastrointestinal reactions are gradually adapted to. These may be ascribed to spasms of smooth muscle caused by *Tripterygium* agent-based irritation of the gastrointestinal mucosa [39]. However, this kind of adverse event was tolerable; after continuing medication or drug withdrawal, it disappeared spontaneously. It has been reported that impairment of the reproductive system is the second most common adverse event, with an incidence rate of 16.7% [37]. During treatment, 16 female patients suffered menstrual disorder, which may result from dysfunction of hypothalamic-hypophyseal-ovarian axis [40]. TwHF and *Tripterygium* agents have abilities to inhibit formation of the corpus luteum and follicles, suppress luteal function and ovulation, and to decrease the levels of estrogen and progesterone; consequently, they may reduce ovarian function [41]. For males, lowered sperm motility is the most common side effect. After discontinuation of the drug, damage to the reproductive system can be restored.

In addition, some included trials reported abnormal liver function after administration of TwHF or *Tripterygium* agents. Although these results were not statistically significant, damage to liver function cannot be ignored. Three trials reported two patients with elevated transaminases, whereas three patients showed slightly abnormal liver function. Lipid production and peroxidation in the liver, induced by TwHF, may be related to this type of adverse event [42].

Currently, numerous methods of alleviating toxic effects have been suggested, such as compatibility with medicines, use of low-dose medication over the long term, and stronger prevention measures [43, 44]. Once adverse events appear, patients should discontinue medication immediately, and clinicians should perform measures to manage adverse events when necessary.

There are also some limitations of this systematic review: (1) the quality of the included trials was not very high. Of the 21 trials, five were RCTs and another five were quasi-randomized controls; the remaining trials simply mentioned “random.” As for allocation concealment and blinding method, only one study described these aspects in detail. Thus, there appeared to be a high risk of selection and detection bias; (2) the sample size was insufficient to reach a robust conclusion; (3) the very low number of events (several subgroup analysis was included in only one study in Figs. 4, 5 and 6) on which the results were based was another limitation that can affect the interpretation of results; (4) the funnel plot was asymmetrical, suggesting a risk of publication bias.

## Conclusion

To summarize, the combination of TwHF or *Tripterygium* agents with antihistamines may be effective for chronic urticaria. However, adverse events should always be noted. The compatibility of medicines, use of low-dose medication over the long term, and the strength of preventive approaches are appropriate methods to reduce toxicity. Furthermore, the study of this treatment requires large sample, multi-center design, and high-quality clinical trials to ascertain its usage in the broader medical field.

## Additional file

Additional file 1: **Table S1.** PRISMA checklist. (DOC 69 kb)

## Abbreviations

ADE: Adverse Events
CI: confidence interval
DLQI: Dermatology Life Quality Index
GTT: Glucosidorum Tripterygll Totorum
LFS: Four-level Score Method
MD: Mean Difference
RER: Recurrence Rate
RR: Risk Ratio
SA: Symptom Assessment
SMD: Standard Mean Difference
SSRI: Symptom Score Reduce lndex
TER: Total Effective Rate
TG: Tripterygium Glycosides
THH: *Tripterygium hypoglaucum* (levl.) Hutch
TwHF: *Tripterygium wilfordii* Hook F
UAS: Urticaria Activity Score
VAS: Visual Analogue Scales
